# Large Right Ventricular Fibroma in a 6-Month-Old Infant

**DOI:** 10.1007/s00246-012-0390-9

**Published:** 2012-05-29

**Authors:** Alice Horovitz, Irene E. van Geldorp, François Roubertie, Jean-Benoit Thambo

**Affiliations:** 1 Department of Congenital Heart Disease, Bordeaux University Hospital, 1, Avenue de Magellan, 33600 Pessac, France; 2 Cardiovascular Research Institute Maastricht, Maastricht University, Maastricht, The Netherlands; 3 Department of Cardiothoracic Surgery, Bordeaux University Hospital, Pessac, France

**Keywords:** Cardiac tumor, Fibroma, Ventricular tachycardia

## Abstract

This report describes the case of a 6-month-old girl with a large cardiac fibroma in the right ventricle. Ventricular tachycardia associated with the fibroma was successfully treated with amiodarone. At the age of 3 years, surgical resection was indicated because of right ventricular outflow tract obstruction caused by progression of the tumor. The fibroma was successfully resected, and further follow-up evaluation was uneventful.

Primary cardiac tumors in children are rare. The incidence is reported to be 0.03–0.32%, with fibroma accounting for approximately 25 % [3]. Fibromas, usually single and large, are most commonly found in the left ventricular free wall or septum and less commonly involve the right ventricle (RV) or atria. We report a case of a young girl with a large cardiac fibroma in an atypical location.

## Case Report

**Fig. 1:**
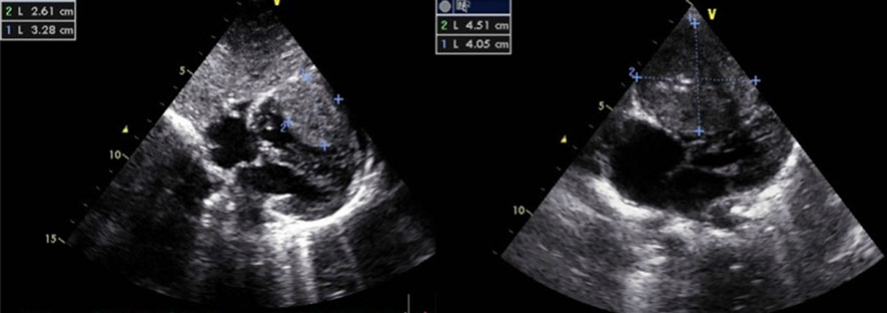
Transthoracic echocardiography showing a large homogeneous mass originating from the right ventricular free wall

**Fig. 2:**
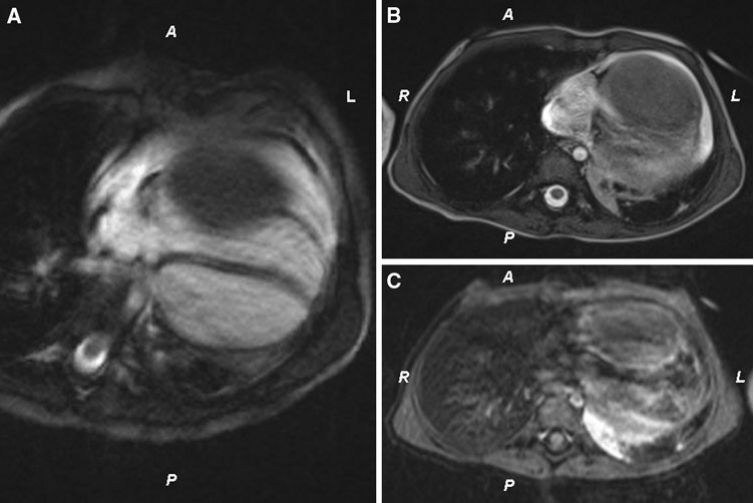
The presence of a solid tumor confirmed by magnetic resonance imaging

In a 6-month-old girl, a large cardiac tumor in the RV was discovered during evaluation for a soft systolic murmur. Except for the soft systolic heart murmur, the overall pediatric examination was normal. Echocardiography showed a large (4.0 × 4.5 × 4.0 cm) homogeneous mass appended to the RV free wall (Fig. 1). There was neither significant RV outflow tract obstruction nor stenosis of the tricuspid valve. Cardiac computed tomography (CT) and magnetic resonance imaging (MRI) confirmed the presence of a large intraventricular solid mass developed at the expense of the RV lateral wall (Fig. 2). Short-term follow-up assessment was complicated by an episode of symptomatic ventricular tachycardia, which was successfully treated with electric cardioversion and amiodarone. Oral therapy with amiodarone was continued under close echocardiographic and rhythmic monitoring with uneventful follow-up evaluation for 2 years.

**Fig. 3:**
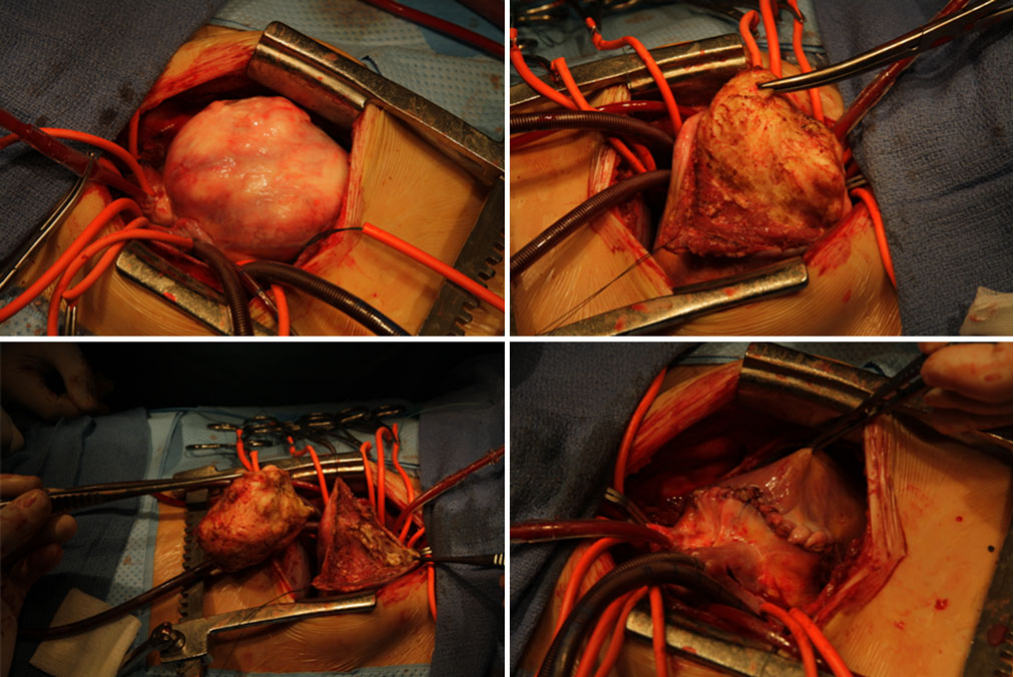
Macroscopy of the encapsulated tumor during the different stages of surgical resection and at its total resection (*lower right panel*)

At the age of 3 years, surgery was clinically indicated because tumor progression had resulted in obstruction of the RV outflow tract. The tumor was completely resected, and the resection margins were plicated (Fig. 3). Histologic examination of an intraoperative tumor biopsy showed abundant proliferation of fibroblasts with collagen deposition, which confirmed the diagnosis of cardiac fibroma.

The girl’s postoperative course was uneventful. Because arrhythmia did not occur during a 6-month follow-up period, the amiodarone treatment was discontinued. Long-term follow-up evaluation was without complications and showed normal cardiac function without recurrence of the tumor.

## Discussion

Although fibromas are histologically benign, they can induce serious cardiovascular complications, including intracavitary obstruction and rhythm disturbances, as illustrated by the reported case and described in the literature [3]. Although treatment of symptomatic fibromas consists of complete excision [4], the management of asymptomatic fibromas remains controversial. Surgery may be complicated by tumor location and size but is reported to result in extended symptom-free survival with acceptable low operative morbidity and mortality, even for patients with large fibromas [1].

Fibromas are not likely to decrease [2], and because they often are associated with clinically significant and potentially life-threatening arrhythmias [3], we think patients should be considered for early surgical treatment when cardiac tumors, probably fibromas, are diagnosed. However, a more pragmatic strategy may be preferred in cases of uncertainty concerning the nature of the tumor or its cleavage plane, when excision of the tumor might affect the integrity of the ventricle, or when individual contraindications for surgery exist. In the reported case, surgery was postponed by treatment of ventricular tachycardia and careful monitoring. For small children, the latter strategy may increase the chances for successful and uncomplicated surgery.
